# The effect of the look-back period for estimating incidence using administrative data

**DOI:** 10.1186/s12913-020-5016-y

**Published:** 2020-03-04

**Authors:** Mira Kim, Kyung-Hee Chae, Youn-Jee Chung, HyeJin Hwang, MinKyung Lee, Hyun-Kyung Kim, Hyun-Hee Cho, Mee-Ran Kim, Chai-Young Jung, Sukil Kim

**Affiliations:** 1 Department of Preventive Medicine, College of Medicine, The Catholic University of Korea, 222, Banpo-daero, Seocho-gu, Seoul, Republic of Korea 06591; 2 Department of Obstetrics and Gynecology, College of Medicine, The Catholic University of Korea, Seoul, Republic of Korea; 3 Biomedical Research Institute, Inha University Hospital, Incheon, Republic of Korea

**Keywords:** Adenomyosis, Administrative data, Endometriosis, Incidence, Look-back period, Misclassification, Uterine leiomyoma

## Abstract

**Background:**

The look-back period is needed to define baseline population for estimating incidence. However, short look-back period is known to overestimate incidence of diseases misclassifying prevalent cases to incident cases. The purpose of this study is to evaluate the impact of the various length of look-back period on the observed incidences of uterine leiomyoma, endometriosis and adenomyosis, and to estimate true incidences considering the misclassification errors in the longitudinal administrative data in Korea.

**Methods:**

A total of 319,608 women between 15 to 54 years of age in 2002 were selected from Korea National Health Insurance Services (KNHIS) cohort database. In order to minimize misclassification bias incurred when applying various length of look-back period, we used 11 years of claim data to estimate the incidence by equally setting the look-back period to 11 years for each year using prediction model. The association between the year of diagnosis and the number of prevalent cases with the misclassification rates by each look-back period was investigated. Based on the findings, prediction models on the proportion of misclassified incident cases were developed using multiple linear regression.

**Results:**

The proportion of misclassified incident cases of uterine leiomyoma, endometriosis and adenomyosis were 32.8, 10.4 and 13.6% respectively for the one-year look-back period in 2003. These numbers decreased to 6.3% in uterine leiomyoma and − 0.8% in both endometriosis and adenomyosis using all available look-back periods (11 years) in 2013.

**Conclusion:**

This study demonstrates approaches for estimating incidences considering the different proportion of misclassified cases for various length of look-back period. Although the prediction model used for estimation showed strong R-squared values, follow-up studies are required for validation of the study results.

## Background

### Health insurance claims as a big data

Administrative data in healthcare primarily refer to the vast medical information available in the form of electronic health records through administrative or health claims data [1]. As the availability of digitized administrative records are increasing, health researchers are able to use these large longitudinal cohort datasets to estimate epidemiologic indicators, such as the incidence and prevalence of various conditions [2–15]. The strengths of this type of large population studies include having a large sample size and avoiding selection or participation bias [16].

The Korean National Health Insurance Service (KNHIS) covers majority of the population as a single payer reimbursing both public and private institutions. All clinics and hospitals submit health insurance claims to the Health Insurance Review and Assessment Service (HIRA) for the claims review each month. The insurance claims include diagnoses (as defined by the International Classification of Diseases 10th revision, ICD-10), demographic information, and medical charges. KNHIS and HIRA share the claims database which represent the entire Korean population and is a major strength in ensuring its applicability for epidemiologic and disease research.

### Estimation of incidence rates from administrative data

Estimating incidence provides a foundation for epidemiologic research, data for resource allocation in health care services, and valuable information for disease prevention. The incidence rate is defined as the ratio of new cases to the total population at risk of the disease. However, the identification of new cases from the administrative data is difficult due to the limited information of patient’s disease status prior to the observatory time span of the data.

A common procedure in determining the incident cases is to exclude cases with the respective diagnoses during the look-back period. A long look-back period allows us to identify more accurate incident cases than a short look-back period. But with a long look-back period, valuable data is lost for analyses. A short look-back period, on the other hand, carries the risk of misclassifying prevalent and recurrent cases as incident cases [17, 18].

Studies have used various time lengths for look-back period [19–22]. Typically, studies have used 3 to 10 year look-back period [19–22] because a look-back period of less than 3 years can lead to extremely overestimated incidences [23]. However, due to limited data, numerous studies have not considered a look-back period or reported a diagnosis-free interval of 1, 2, or 3 years [24–27]. Additionally, most of studies focused on the estimated the one- year incidence by applying different look-back period [28–30], and there were few studies investigating the incidence trend in longitudinal data. In this study, we intended to investigate the incidence trend considering the increasing look-back period every year in the longitudinal administrative data.

The purposes of this study are to evaluate the impact of various look-back period on the observed incidences of uterine leiomyoma, endometriosis and adenomyosis which are the most common gynecologic diseases in reproductive women and associated with the infertility and adverse pregnancy outcomes [31], and to estimate the true incidences with their trends considering the misclassification error rates using the longitudinal administrative health data in South Korea. While it is advisable to have a sufficiently long look-back period when calculating the incidence using administrative data, we sought a way to minimize data loss.

## Methods

### Data source

We conducted a retrospective population-based cohort study using the National Health Insurance Service–National Sample Cohort (NHIS-NSC) 2002–2013. The data were produced by the KNHIS using a systematic sampling method to generate a representative sample from the target population of 46,605,433 individuals in 2002. The database is comprised of 1,025,340 subjects which accounts for approximately 2.2% of the total eligible Korean population in the year of 2002 who were followed up for 11 years until 2013. The representativeness of the data had been presented elsewhere [32].

It is a semi-dynamically constructed cohort database with individuals that have been followed up to the time of death, emigration, or until the end of the study period and addition of newborn infants included into the database annually [32]. This database includes all medical claims filed from January 2002 to December 2013. More details of the cohort are described elsewhere [32].

### Selection of subjects

Patients in Korea tend to visit several healthcare institutions for any reason, as the patients can access clinics, specialists, and hospitals without restriction. Thus, it is possible for a patient to visit several clinics/hospitals in one day, has multiple diagnostic codes at a time, has multiple claims on the same day in the same clinic/hospital, or has both outpatient treatment and hospital admission on the same day. Therefore, one claim should be selected to define incidence in consideration of all these cases. We set priorities in the following order.

First, priority is given to the claim with the earlier hospital visit date. If there are several patients who visited hospital on same date, inpatient’s statement takes priority over outpatient’s one. Among several outpatient statements, a statement with a high ranking of diagnosis codes is selected in ascending order. If the order of the diagnostic codes is the same, priority is given to that with higher medical costs. Finally, priority is given to the one with earlier billing number. Even though individuals have some gaps of few years between 2002 and 2013 in the record, we considered they are continually insured patients and included in the subject.

**Fig. 1:**
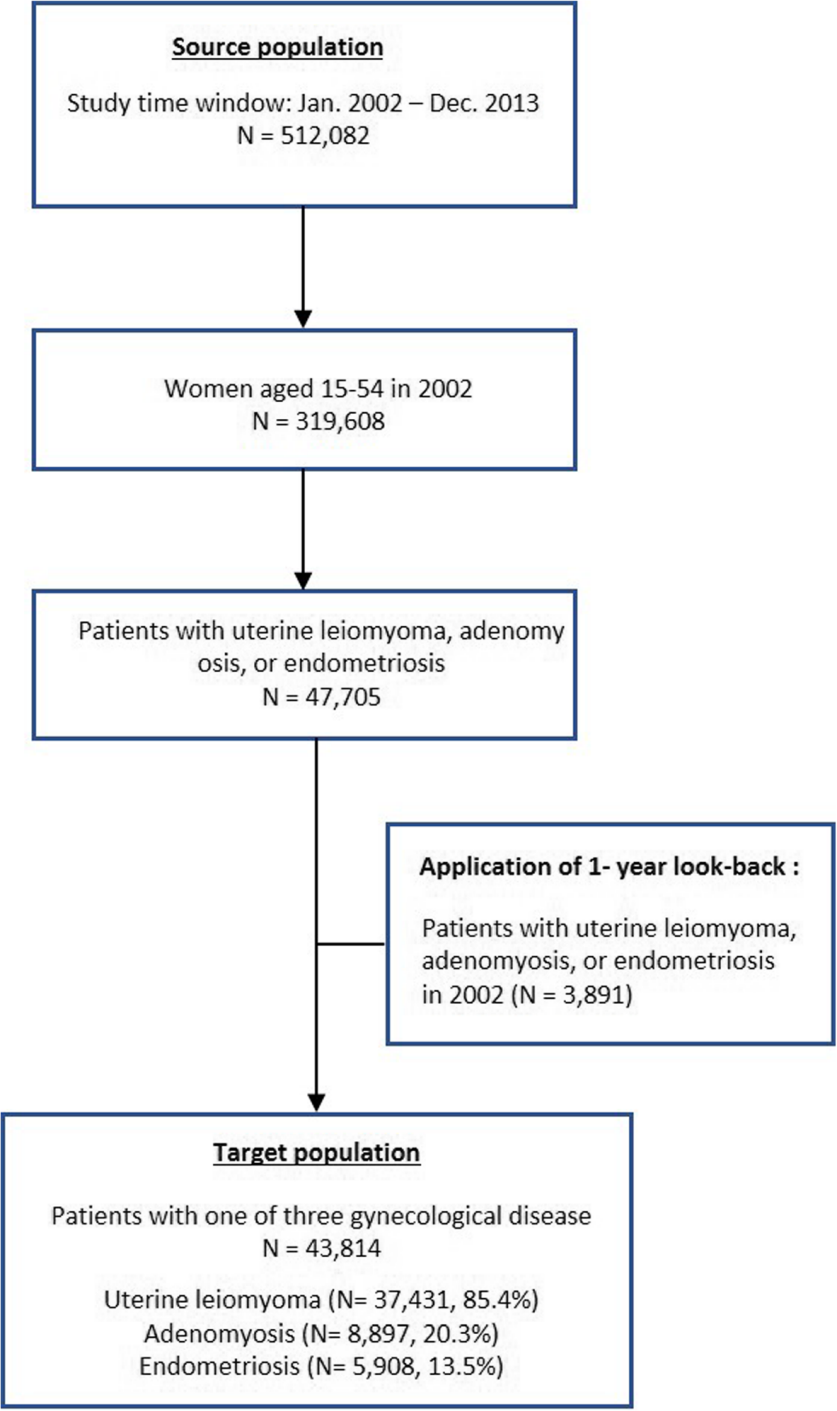
Flow chart of case identification

A flow chart indicating the number of patients with one of three gynecological diseases is shown in Fig. 1. The population denominator was a total of 319,608 women aged 15–54 who were eligible for the National Health Insurance in 2002 among 512,082 female individuals from the KNHIS cohort database. Those women were followed up for 11 years until 2012. The incident cases were defined using the standardized codes from the Korean version of the International Classification of Diseases 10th Edition (ICD-10). Cases with diagnostic codes of the target diseases coded in the health insurance claims between 2002 and 2013 regardless of service types were identified; The target diseases of interest were uterine leiomyoma (ICD-10: D25, D25.0, D25.1, D25.2, D25.9), adenomyosis (ICD-10: N80.0), and endometriosis (ICD-10: N80, N80.1, N80.2, N80.3, N80.4, N80.5, N80.6, N80.8, N80.9).

To identify the patients with prior history of the disease, one-year look-back period as of 2003 was applied at the discretion of obstetricians and gynecologists that patients would visit the gynecologists within one year after the onset of diseases. There were 43,814 patients after excluding patients with the target diseases in 2002. Patients who had concurrent diagnoses of uterine leiomyoma, adenomyosis, or endometriosis were counted in each of the targeted disease. Therefore, there were 37,431 patients with a diagnosis for uterine leiomyoma, 8897 for adenomyosis, and 5908 for endometriosis.

### Estimated incidence

To assess the relationship between the look-back period and the number of misclassified cases, the annual number of patients diagnosed with either uterine leiomyoma, adenomyosis, or endometriosis (prevalent cases) from 2003 to 2013 were determined, and the number of prevalent cases misclassified as incident cases were identified with increasing look-back period by each observation year (Additional File 1).

The association between the year of diagnosis and the number of prevalent cases with the misclassification rates by each look-back period was investigated. Based on the findings, prediction models on the proportion of misclassified incident cases were developed using multiple linear regression. The model of best fit was selected by using the lowest root mean square error (RMSE) or the largest adjusted R-squared value, which are good measures of assessing the accuracy of prediction model. Estimated incidences were calculated using the best prediction model and compared with the observed incidences.

## Results

### Misclassification rates of each year by different length of look-back period

The Table 1 shows the number of prevalent cases with uterine leiomyoma in each year. The number of prevalent cases in 2003 was 3092 and continued to increase by year. By 2013, the number of prevalent cases increased to 8348, which was twice the number of prevalent cases from 2003. Look-back period of each observation year were determined by increasing the look-back period by one year from 2003 (i.e. 2003 had up to 1 one-year look-back period, whereas 2013 had up to 11-year look-back period). With adding more years of look-back period, the proportion of prevalent cases misclassified as incident cases increased.

**Table 1:** The number of prevalent cases detected by various lengths of look-back period each year (2003–2013) for uterine leiomyoma (%^1^)

Year	Prevalent cases (*n*)	Look-back period (years)											Estimated cases (*n*, %)^2^
		1	2	3	4	5	6	7	8	9	10	11	
2003	3902	785 20.1											1808 46.3
2004	4475	1065 23.8	1282 28.6										2122 47.4
2005	5042	1208 24.0	1554 30.8	1710 33.9									2445 48.5
2006	5347	1417 26.5	1840 34.4	2047 38.3	2151 40.2								2650 49.6
2007	5828	1564 26.8	2056 35.3	2276 39.1	2399 41.2	2460 42.2							2951 50.6
2008	5843	1603 27.4	2067 35.4	2293 39.2	2423 41.5	2498 42.8	2556 43.7						3021 51.7
2009	6369	1795 28.2	2397 37.6	2726 42.8	2895 45.5	3008 47.2	3090 48.5	3135 49.2					3361 52.8
2010	6930	2048 29.6	2597 37.5	2941 42.4	3139 45.3	3250 46.9	3341 48.2	3410 49.2	3454 49.8				3732 53.8
2011	7313	2244 30.7	2811 38.4	3105 42.5	3326 45.5	3455 47.2	3561 48.7	3630 49.6	3675 50.3	3698 50.6			4016 54.9
2012	8125	2542 31.3	3253 40.0	3632 44.7	3829 47.1	4013 49.4	4130 50.8	4210 51.8	4257 52.4	4303 53.0	4338 53.4		4549 56.0
2013	8348	2661 31.9	3350 40.1	3750 44.9	3966 47.5	4138 49.6	4248 50.9	4338 52.0	4402 52.7	4459 53.4	4502 53.9	4522 54.2	4764 57.1

^1^ Prevalent cases detected by look-back period divided by prevalent cases

^2^ Estimated misclassified cases (*n*) and the misclassification rate (%) for 11 years look-back period calculated using the prediction model

The grey cells at the last column of each observation year show the number of prevalent cases misclassified as incident cases which were discovered by applying the look-back period (Table 1). In 2003 with a one-year look-back period, among a total of 3902 patients with uterine leiomyoma, there were 785 (20.1%) cases that were misclassified as incident cases. In 2013, however, with 11 years of look-back period among 8348 cases, the misclassified as incident cases increased to 4522 (54.2%).

Tables 2 and 3 show the proportion of patients diagnosed with adenomyosis and endometriosis and misclassified as incident cases for each look-back period. With a look-back period of 11 years, 733 (41.6%) patients with adenomyosis and 494 (50.3%) patients with endometriosis were estimated to have prior history of the disease.

**Table 2:** The number of prevalent cases detected by various lengths of look-back period in each year (2003–2013) for adenomyosis (%^1^)

Year	Prevalent cases (*n*)	Look-back period (years)											Estimated cases (*n*, %)^2^
		1	2	3	4	5	6	7	8	9	10	11	
2003	650	54 8.3											116 17.8
2004	648	77 11.9	83 12.8										130 20.1
2005	849	105 12.4	130 15.3	140 16.5									191 22.5
2006	823	111 13.5	143 17.4	155 18.8	161 19.6								204 24.8
2007	925	162 17.5	190 20.5	202 21.8	211 22.8	218 23.6							251 27.1
2008	1010	170 16.8	204 20.2	225 22.3	234 23.2	238 23.6	240 23.8						298 29.5
2009	1195	241 20.2	287 24.0	309 25.9	332 27.8	340 28.5	348 29.1	352 29.5					380 31.8
2010	1393	331 23.8	397 28.5	422 30.3	444 31.9	456 32.7	464 33.3	471 33.8	473 34.0				476 34.2
2011	1590	367 23.1	437 27.5	471 29.6	490 30.8	507 31.9	519 32. 6	524 33.0	530 33.3	530 33.3			580 36.5
2012	1664	444 26.7	533 32.0	573 34.4	600 36.1	609 36.6	617 37 .1	622 37.4	624 37.5	626 37.6	628 37.7		646 38.8
2013	1762	487 27.6	596 33.8	646 36.7	671 38.1	689 39.1	699 39.7	711 40.4	721 40.9	724 41.1	729 41.4	733 41.6	725 41.2

^1^ Prevalent cases detected by look-back period divided by prevalent cases

^2^ Estimated misclassified cases (*n*) and the misclassification rate (%) for 11 years look-back period calculated using the prediction model

**Table 3:** The number of prevalent cases detected by various lengths of look-back period in each year (2003–2013) for endometriosis (%^1^)

Year	Prevalent cases (*n*)	Look-back period (years)											Estimated Cases (*n*, %)^2^
		1	2	3	4	5	6	7	8	9	10	11	
2003	750	127 16.9											212 28.2
2004	770	145 18.8	164 21.3										234 30.4
2005	797	168 21.1	194 24.3	208 26.1									259 32.5
2006	804	194 24.1	216 26.9	230 28.6	236 29.4								279 34.7
2007	738	206 27.9	245 33.2	261 35.4	272 36.9	275 37.3							272 36.9
2008	730	219 30.0	238 32.6	248 34.0	253 34.7	262 35.9	264 36.2						285 39.0
2009	847	231 27.3	264 31.2	275 32.5	290 34.2	304 35.9	309 36.5	311 36.7					349 41.2
2010	959	309 32.2	348 36.3	375 39.1	397 41.4	406 42.3	410 42.8	418 43.6	421 43.9				416 43.4
2011	965	360 37.3	389 40.3	403 41.8	417 43.2	426 44.1	437 45.3	442 45.8	449 46.5	450 46.6			439 45.5
2012	957	350 36.6	385 40.2	404 42.2	411 42.9	423 44.2	429 44.8	431 45.0	433 45.2	439 45.9	442 46.2		457 47.7
2013	983	366 37.2	422 42.9	445 45.3	452 46.0	456 46.4	464 47.2	471 47.9	480 48.8	489 49.7	493 50.2	494 50.3	490 49.9

^1^ Prevalent cases detected by look-back period divided by prevalent cases

^2^ Estimated misclassified cases (*n*) and the misclassification rate (%) for 11 years look-back period calculated using the prediction model

### Prediction of the proportions of misclassification

The year of diagnosis and the number of patients were linearly related with the proportion of misclassification for uterine leiomyoma, adenomyosis and endometriosis, and the look-back period was logarithmically related with the proportion of misclassification (Supplementary Fig. 1, 2 and 3). Using these findings, four prediction models were developed (Table 4). Model A was selected as the model of best fit because it had the smallest RMSE and highest estimated R-squared value. The independent variables were the year of diagnosis and the log-transformed look-back period.

**Table 4:** Comparison of the prediction models by RMSE and estimated R^2^

Model	Independent variable		intercept	Regression coefficient^1^		RMSE	Adj R^2^
	X_1_	X_2_		ß_1_	ß_2_		
Uterine leiomyoma							
A	Ln (Look-back)	Year	1	0.0967	0.01072	0.01297	0.9788
B	Ln (Look-back)	Patients size	0.1385	0.09757	0.00002347	0.01387	0.9757
C	Look-back	Year	−22.6134	0.0233	0.01141	0.03295	0.8632
D	Look-back	Patients size	0.14922	0.02358	0.00002459	0.03375	0.8565
*Adenomyosis*							
A	Ln (Look-back)	Year	−46.7434	0.0464	0.02337	0.01371	0.9745
B	Ln (Look-back)	Patients size	−0.00698	0.04887	0.00017117	0.01637	0.9636
C	Look-back	Year	−47.0381	0.01154	0.02352	0.01877	0.9522
D	Look-back	Patients size	0.000494	0.01203	0.00017177	0.02195	0.9346
*Endometriosis*							
A	Ln (Look-back)	Year	1.83882	0.0034	0.00091597	0.01762	0.9549
B	Ln (Look-back)	Patients size	−0.12183	0.0609	0.00047936	0.03409	0.8312
C	Look-back	Year	−43.6181	0.01176	0.02187	0.02229	0.9278
D	Look-back	Patients size	−0.10688	0.01539	0.0004722	0.03793	0.7911

^1^ ß1 and ß2 are the regression coefficients of independent variable X_1_ and X_2_

### Estimated number of incident cases

Table 5 shows the number of observed and estimated incident cases per year. The proportions of misclassified cases of uterine leiomyoma, adenomyosis and endometriosis were 32.8, 10.4 and 13.6%, respectively in 2003 with one-year look back period. The proportions of misclassified cases of uterine leiomyoma in 2003 was about 3 times that of adenomyosis and endometriosis. The proportions of misclassified cases decreased to 6.3% in uterine leiomyoma, − 0.8% in both adenomyosis and endometriosis in 2013 with 11 years of look-back period.

**Table 5:** The proportions of misclassified between observed incident cases and estimated incident cases

Incidence	Look-back period (years)										
	2003 (1)	2004 (2)	2005 (3)	2006 (4)	2007 (5)	2008 (6)	2009 (7)	2010 (8)	2011 (9)	2012 (10)	2013 (11)
*Uterine leiomyoma*											
Observed (*n*)	3117	3193	3332	3196	3368	3287	3234	3476	3615	3787	3826
Estimated (*n*)	2094	2353	2597	2697	2877	2822	3008	3198	3297	3576	3584
Proportions of misclassified (*%*)	32.8	26.3	22.1	15.6	14.6	14.1	7.0	8.0	8.8	5.6	6.3
*Adenomyosis*											
Observed	596	565	709	662	707	770	843	920	1060	1036	1029
Estimated	534	518	658	619	674	712	815	917	1010	1018	1037
Proportions of misclassified (*%*)	10.4	8.3	7.2	6.5	4.7	7.5	3.3	0.3	4.7	1.7	−0.8
*Endometriosis*											
Observed	623	606	589	568	463	466	536	538	515	515	489
Estimated	538	536	538	525	466	445	498	543	526	500	493
Proportions of misclassified (*%*)	13.6	11.6	8.7	7.6	−0.6	4.5	7.1	−0.9	−2.1	2.9	−0.8

## Discussion

Administrative health claims database was used to calculate the annual incident cases of uterine leiomyoma, adenomyosis and endometriosis in South Korea (2003–2013). The proportion of misclassified prevalent cases as incident cases was estimated according to various length of look-back period in years. As the look-back period increased, the proportion of misclassified incident cases decreased. Shorter look-back period incurred incidences with greater proportion of misclassification.

It is difficult to accurately identify new cases in patients diagnosed each year because misclassification bias exists in which the prevalent case is considered as an incidence case according to look-back period changing every year during the research period. Thus, to minimize this systematic error, we used 11 years of claim data to estimate the incidence by equally setting the look-back period to 11 years for each year using prediction model.

### Optimal look-back period for annual incidence

As mentioned in the Abbas’s study, the optimal look-back period for annual incidence while minimizing the rate of misclassification depended on the nature and the stage of the respective diseases [23]. In uterine leiomyoma and adenomyosis, the proportion of misclassified cases decreased by about 50% when the look-back period increased from 6 years to 7 years, and in endometriosis, it decreased by about 10% when the look-back period increased from 7 years to 8 years. The proportion of misclassified cases of endometriosis in 2007 is − 0.6 which is considerably smaller than 7.6, the rate of previous year. Therefore, disease-specific look-back period required at least 7 years for uterine leiomyoma and adenomyosis, and 8 years for endometriosis.

The extent of misclassification varies by diseases even though the same length of look-back period was applied. In 2003 with one-year look-back period, the proportion of misclassification for uterine leiomyoma was 32.8%, while for adenomyosis and endometriosis were 10.4 and 13.6%, respectively. Similarly, in the 11 years of look-back period in 2013, the proportion of misclassification for uterine leiomyoma was 6.3% and − 0.8% for adenomyosis and endometriosis, which is negligible.

Incidences can be affected by external effect. The number of endometriosis patients significantly decreased in 2007, and thereafter did not increase. One possible reason is that the HIRA has strengthened coding requirement to use full digit detail codes in 2006. Subsequently, the number of endometriosis patients with N80 might be redistributed to N80.0 for adenomyosis and N80.1 to N80.9 for the endometriosis. The estimated number of incident cases of the disease in 2013 should be interpreted with caution. When the estimated incidence is lower than the observed incidence, the observed incidence should be used instead of the estimated incidence for the practical use.

According to the Organization for Economic Co-operation and Development (OECD) statistics in 2018, the annual number of outpatient visits per capita in Korea in 2016 was 17.0 which is the highest among OECD countries and 2.5 times more than the OECD average (6.9) [33]. As such, the same duration of look-back period using administrative health data in Korea is estimated to have an increase in the proportion of misclassification than other OECD countries.

### Strengths and limitations

The strengths of this study include large sample size and long observation period of 12 years. This increases the accuracy for calculating the incidences and proportion of misclassifications. However, the study has several limitations.

In the regression model for estimating the number of incident cases, a linear function for the observation year and a log function for the look-back was used. There were 11 data points for the one-year look-back, but only one point for the 11-year look-back. Although the prediction model had a good RMSE and R-squared, the model was based on uneven distribution of the observed data points may adversely affected the fit of the model.

The study has inherent limitations as this study was based on secondary data analyses of the NHIS cohort database. We could not definitely confirm the diagnosis codes for every single patient in the database since the diagnostic code of the claim data alone cannot guarantee the accuracy of the diagnosis [34]. According to Park et al. [35], about 70% of primary diagnosis codes concurred with medical records. Issues concerning studies involving administrative data are well described in Mazzali, C. and and P. Duca’s study [36]. When the cases were confirmed by prescription codes and procedure code in addition to the diagnostic codes, the incidences would be lower than this study results. Lastly, asymptomatic and/or undiagnosed patients cannot be detected using the health claims data. This would decrease the proportion of the true incident cases of the diseases.

## Conclusion

Using the NHIS administrative heath database, various length of the look-back period was applied to estimate the incidences of uterine leiomyoma, adenomyosis, and endometriosis and determine the different proportion of misclassification errors for each look-back period. The prediction model was used to adjust the misclassification errors that occur when calculating incidence trend derived from longitudinal administrative data. Although the prediction model used for estimation showed strong R-squared values, follow-up studies are required for validation of the study results.

In the longitudinal data, the look-back period applied for incidence estimation generated different misclassification errors for each look-back period. We proposed a method to adjust the misclassification errors when calculating the incidence using administrative data. Even though we focused on the three gynecological disease in this study, the approaches presented in this study are applicable to other diseases as well.

## Supplementary information

**Additional file 1.** A detailed description of model construction.

**Additional file 2: Supplementary Figure S1.** The number of prevalent cases and misclassification rate detected by various lengths of the look-back period per year between 2003 and 2013 forwomen with uterine leiomyoma.

**Additional file 3: Supplementary Figure S2.** The number of prevalent cases and misclassification rate detected by various lengths of the look-back period per year between 2003 and 2013 for women with adenomyosis.

**Additional file 4: Supplementary Figure S3.** The number of prevalent cases and misclassification rate detected by various lengths of the look-back period per year between 2003 and 2013 for women with endometriosis.

## Data Availability

The data that support the findings of this study are not publicly available. Data are however available from the Korea National Health Insurance Service (KNHIS) upon request and with permission of KNHIS.

## Abbreviations

HIRA: Health Insurance Review and Assessment Service
ICD-10: International Classification of Diseases 10th revision
KNHIS: Korean National Health Insurance Service
NHIS-NSC: National Health Insurance Service–National Sample Cohort
OECD: Organization for Economic Co-operation and Development
RSME: Root mean square error

## References

[1] Trifiro G, Sultana J, Bate A. From big data to smart data for Pharmacovigilance: the role of healthcare databases and other emerging sources. Drug Saf. 2017. 10.1007/s40264-017-0592-4 28840504

[2] Gothe H (2016). The use of administrative data to determine prevalence and incidence of Copd: a systematic review. Value Health.

[3] Widdifield J (2015). Development and validation of an administrative data algorithm to estimate the disease burden and epidemiology of multiple sclerosis in Ontario, Canada. Multiple Scler.

[4] Ng, R., et al. Brain disorders in Ontario: prevalence, incidence and costs from health administrative data*.* Institute for Clinical Evaluative Sciences: Toronto, Ontario, 2015.

[5] Nigwekar SU (2014). Quantifying a rare disease in administrative data: the example of calciphylaxis. J Gen Intern Med.

[6] Benchimol EI (2014). Validation of international algorithms to identify adults with inflammatory bowel disease in health administrative data from Ontario, Canada. J Clin Epidemiol.

[7] Ward MM (2013). Estimating disease prevalence and incidence using administrative data: some assembly required. J Rheumatol.

[8] Marrie RA (2012). The incidence and prevalence of thyroid disease do not differ in the multiple sclerosis and general populations: a validation study using administrative data. Neuroepidemiology.

[9] Floyd JS (2012). Use of administrative data to estimate the incidence of statin-related rhabdomyolysis. JAMA.

[10] Benchimol EI (2009). Increasing incidence of paediatric inflammatory bowel disease in Ontario, Canada: evidence from health administrative data. Gut.

[11] Mirkin D, Murphy-Barron C, Iwasaki K (2007). Actuarial analysis of private payer administrative claims data for women with endometriosis. J Manag Care Pharm.

[12] Bernatsky S (2007). A population-based assessment of systemic lupus erythematosus incidence and prevalence—results and implications of using administrative data for epidemiological studies. Rheumatology.

[13] Segal J, Powe N (2006). Prevalence of immune thrombocytopenia: analyses of administrative data. J Thromb Haemost.

[14] Hux JE (2002). Diabetes in Ontario: determination of prevalence and incidence using a validated administrative data algorithm. Diabetes Care.

[15] Hamad R (2015). Using "big data" to capture overall health status: properties and predictive value of a claims-based health risk score. PLoS One.

[16] Chen YC (2011). Reduced access to database. A publicly available database accelerates academic production. BMJ.

[17] Czwikla J, Jobski K, Schink T (2017). The impact of the lookback period and definition of confirmatory events on the identification of incident cancer cases in administrative data. BMC Med Res Methodol.

[18] Sulo G (2015). Effect of the Lookback Period's length used to identify incident acute myocardial infarction on the observed trends on incidence rates and survival: cardiovascular disease in Norway project. Circ Cardiovasc Qual Outcomes.

[19] Yeh RW (2010). Population trends in the incidence and outcomes of acute myocardial infarction. N Engl J Med.

[20] Koopman C (2013). Population trends and inequalities in incidence and short-term outcome of acute myocardial infarction between 1998 and 2007. Int J Cardiol.

[21] Salomaa V (2007). Use of secondary preventive medications after the first attack of acute coronary syndrome. Eur J Cardiovasc Prev Rehabil.

[22] Lin JC, Shau WY, Lai MS (2014). Sex- and age-specific prevalence and incidence rates of sight-threatening diabetic retinopathy in Taiwan. JAMA Ophthalmol.

[23] Abbas S (2012). Estimation of disease incidence in claims data dependent on the length of follow-up: a methodological approach. Health Serv Res.

[24] Boehme MW (2015). Prevalence, incidence and concomitant co-morbidities of type 2 diabetes mellitus in South Western Germany--a retrospective cohort and case control study in claims data of a large statutory health insurance. BMC Public Health.

[25] Seo HJ, Oh IH, Yoon SJ (2012). A comparison of the cancer incidence rates between the national cancer registry and insurance claims data in Korea. Asian Pac J Cancer Prev.

[26] Kim H (2016). Estimating epilepsy incidence and prevalence in the US pediatric population using Nationwide health insurance claims data. J Child Neurol.

[27] Linsell L (2006). Prevalence and incidence of adults consulting for shoulder conditions in UK primary care; patterns of diagnosis and referral. Rheumatology (Oxford).

[28] Schmedt N (2017). Incidence of multiple sclerosis in Germany: a cohort study applying different case definitions based on claims data. Neuroepidemiology.

[29] Smolina K (2012). Incidence and 30-day case fatality for acute myocardial infarction in England in 2010: national-linked database study. Eur J Public Health.

[30] Worthington JM (2017). Differentiating incident from recurrent stroke using administrative data: the impact of varying lengths of look-Back periods on the risk of misclassification. Neuroepidemiology.

[31] Wise LA, Laughlin-Tommaso SK (2016). Epidemiology of uterine fibroids: from menarche to menopause. Clin Obstet Gynecol.

[32] Lee J (2017). Cohort profile: the National Health Insurance Service-National Sample Cohort (NHIS-NSC)*, South Korea*. Int J Epidemiol.

[33] OECD. *OECD Health Statistics* 2018. [cited 2018 Sep. 3]; Available from: http://www.oecd.org/els/health-systems/.

[34] Sohn S (2016). A nationwide epidemiological study of newly diagnosed spine metastasis in the adult Korean population. Spine J.

[35] Park BJ, Park PK, sung KH. Validity of diagnosis code on National Health Insurance Claim Database. Seoul: Seoul National University School of Medicine; 2003.

[36] Mazzali C, Duca P (2015). Use of administrative data in healthcare research. Intern Emerg Med.

