# Supplementary material for: The effect of the look-back period for estimating incidence using administrative data
Source: BMC Health Serv Res. 2020 Mar 4;20:166. doi: 10.1186/s12913-020-5016-y (PMC7057623; doi:10.1186/s12913-020-5016-y)
Supplement: Supplementary file 1 — Additional file 1. A detailed description of model construction. [file 12913_2020_5016_MOESM1_ESM.docx]

**Detailed description of model construction**We conducted multiple linear regression to predict misclassification rate by the look-back period, the number of patients and the year of diagnosis since the misclassification rate of each disease depends on those factors.

The following graphs are the distribution of misclassification rate by the look-back period, the number of patients and the year of diagnosis for uterine leiomyoma. The year of diagnosis (x-axis) and the number of patients (x-axis) were linearly related with the proportion of misclassification (y-axis), and the look-back period (x-axis) was both linearly and logarithmically related with the proportion of misclassification (y-axis). The other diseases showed the same distribution patterns.

Using these findings, we developed four prediction models using covariates of year of diagnosis, number of patients and look-back period, and the Model A with the smallest value of RMSE and highest value of adjusted R squared was selected (Please see Table 4). Thus, the year of diagnosis and the log-transformed look-back period entered into the regression model and estimated the number of misclassification cases in the 11^th^ look-back year for each year.

1. The distribution of misclassification rate for **Uterine Leiomyoma**

|  |
| --- |
|  |
|  |

1. The distribution of misclassification rate for **Adenomyosis**

|  |
| --- |
|  |
|  |

1. The distribution of misclassification rate for **Endometriosis**

|  |
| --- |
|  |
|  |
