# Supplementary figures and images for: The effect of the look-back period for estimating incidence using administrative data
Source: BMC Health Serv Res. 2020 Mar 4;20:166. doi: 10.1186/s12913-020-5016-y (PMC7057623; doi:10.1186/s12913-020-5016-y)

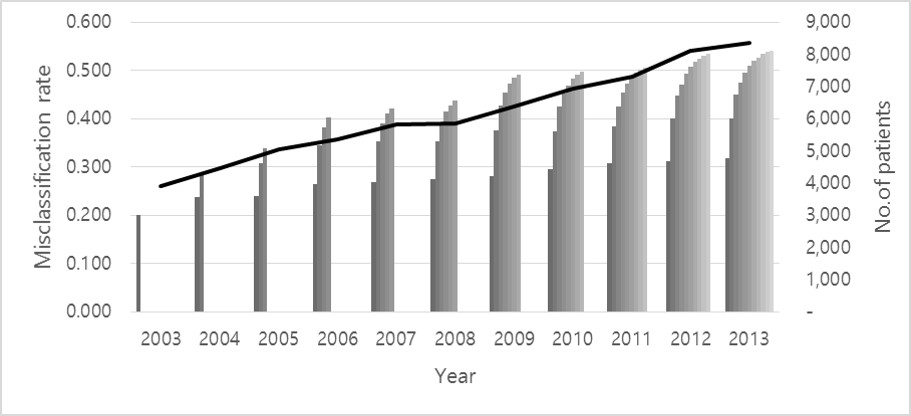

Supplement: Supplementary file 2 — Additional file 2: Supplementary Figure S1. The number of prevalent cases and misclassification rate detected by various lengths of the look-back period per year between 2003 and 2013 forwomen with uterine leiomyoma. [file 12913_2020_5016_MOESM2_ESM.jpg]

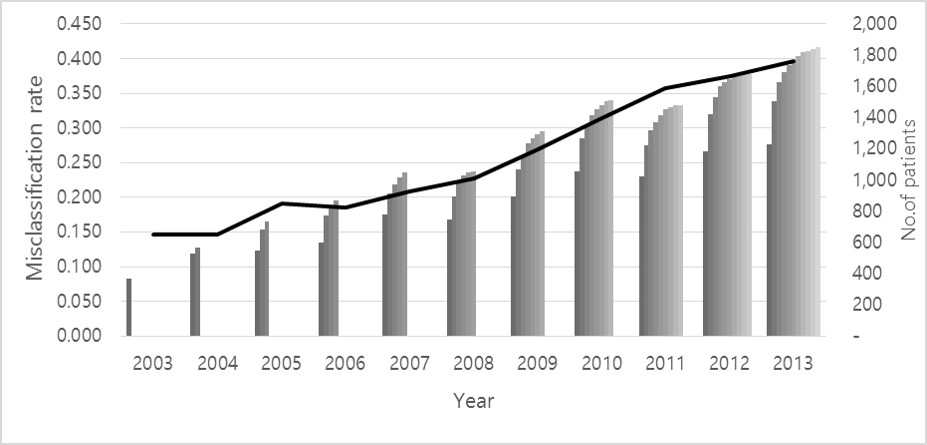

Supplement: Supplementary file 3 — Additional file 3: Supplementary Figure S2. The number of prevalent cases and misclassification rate detected by various lengths of the look-back period per year between 2003 and 2013 for women with adenomyosis. [file 12913_2020_5016_MOESM3_ESM.jpg]

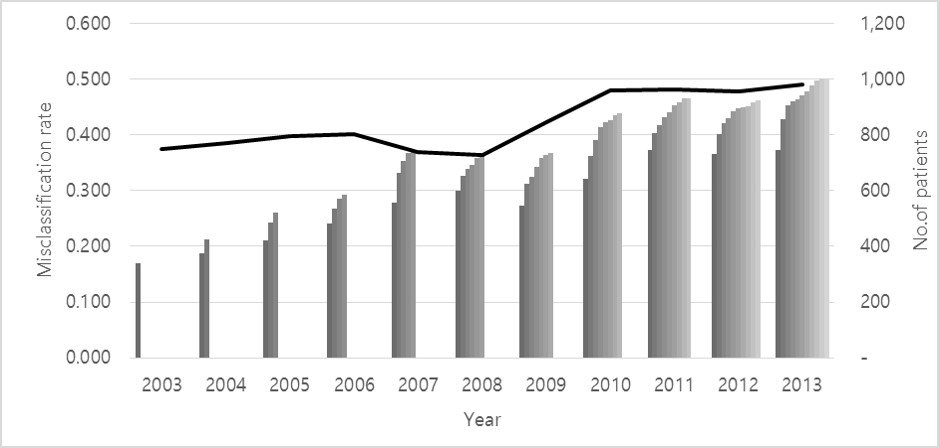

Supplement: Supplementary file 4 — Additional file 4: Supplementary Figure S3. The number of prevalent cases and misclassification rate detected by various lengths of the look-back period per year between 2003 and 2013 for women with endometriosis. [file 12913_2020_5016_MOESM4_ESM.jpg]
